# Mesenchyme Stem Cell-Derived Conditioned Medium as a Potential Therapeutic Tool in Idiopathic Pulmonary Fibrosis

**DOI:** 10.3390/biomedicines10092298

**Published:** 2022-09-16

**Authors:** George Kolios, Vasilis Paspaliaris

**Affiliations:** 1 Lab of Pharmacology, Faculty of Medicine, Democritus University of Thrace, 68100 Alexandroupolis, Greece; 2 Tithon Biotech Inc., San Diego, CA 92127, USA

**Keywords:** Mesenchyme Stem Cells, idiopathic pulmonary fibrosis, Mesenchyme Stem Cell-Derived Conditioned Medium, conditioned medium

## Abstract

Mesenchyme Stem Cells (MSCs) are the most used types of stem cells in regenerative medicine. Regenerative medicine is a rapidly emerging medicine section that creates new methods to regrow, restore, and replace diseased and damaged tissues, organs, and cells. Scholars have shown a positive correlation between MSCs-based therapies and successful treatment of diseases like cardiac ischemia, cartilage problems, bone diseases, diabetes, and even neurological disorders. Although MSCs have several varying features that make them unique, their immuno-regulatory effects in tissue repair emerge from their secretion of paracrine growth factors, exosomes, and cytokines. These cells secrete a secretome, which has regenerative and reparative properties that lead to injury amelioration, immune modulation, or fibrosis reduction. Recent studies have shown that the administration MCSs derived conditioned medium (MSCs-CM) in acute doses in humans is safe and well-tolerated. Studies from animal models and human clinical trials have also shown that they are efficacious tools in regenerative medicine. In this review, we will explore the therapeutic potential of MSCs-CM in pulmonary fibrosis, with further insight into the treatment of Idiopathic Pulmonary Fibrosis (IPF).

## 1. Introduction

Stem cells are cell populations characterized by their ability to self-renew and differentiate into various cell types. Their potency is categorized according to their degree of differentiation [1]. Mesenchyme Stem Cells (MSCs), which are multipotent, are a type of stem cell commonly used in regenerative medicine as, apart from their plasticity and tropism, they have immunomodulatory properties [2]. Although MSCs have several varying features attributed to their physical proximity to the diseased or damaged cells and make them unique [3], their immunoregulatory effects and tissue repair emerge from their secretion of paracrine growth factors, exosomes, and cytokines, which process regenerative and reparative properties leading to injury amelioration, immune modulation, or fibrosis reduction [4,5]. In addition, epithelial-mesenchymal transition is a common downstream mechanism of all fibrotic disorders, but its implication in the development of pulmonary fibrosis has been widely discussed and is still obscure [6]. During the last decade, extensive research at both preclinical and clinical levels has shown that MSCs-CM — containing its secretome—has therapeutic potential in the treatment of several disorders, such as MSCs therapy. The potential to bypass the problems associated with performing live stem cell therapy and effectively putting a stem cell therapy “in a vial” indicates an important therapeutic role of MSCs-CM in regenerative medicine [7].

Idiopathic pulmonary fibrosis (IPF) is a rare pulmonary disease of unknown cause, characterized by inflammation, and the progressive deposition of extracellular matrix that is associated with the formation of scar tissue leading to significant remodeling of the lungs [8,9,10]. IPF is a diffuse parenchymal lung disease (DPLD) [11] and it distinguishes itself from other DLPDs with a unique histological pattern known as usual interstitial pneumonia (UIP) [12]. UIP presents with significant lung volume loss, peripheral septal thickening, bronchiectasis, and a honeycomb pattern of fibrosis [13]. As these develop, patients will experience worsening dyspnea, chronic dry cough, hypoxemia, weight loss, and cardiac issues, such as right ventricular failure [8,14]. By definition of idiopathic, the etiology of IPF is mostly unknown, though several risk factors that may contribute to disease development have been found. Several studies suggest that intrinsic risk factors, including advanced age or being male, extrinsic risk factors such as environmental exposures and cigarette smoking, and co-morbidities, including gastroesophageal reflux disease, may independently increase susceptibility for IPF or act in a synergistic fashion contributing to increased risk for disease development [15,16,17]. Typically occurring later in life, IPF appears mainly in adults older than 50, with a mean age of presentation of 66 years old [10,14]. Although the exact relationship between ageing and IPF is not understood, ageing causes loss of telomeres, epigenetic alterations, cellular senescence, and genomic instability, leading to the development of random cellular and genetic defects [18]. The collagen build-up is a crucial factor in the development of IPF regardless of the source of initial damage. The fibrotic tissue in the lungs is primarily comprised of collagen, meaning that increased levels of collagen are indicative of fibrosis [19]. The standard of care for IPF patients is currently limited to two FDA-approved pharmacological therapies, highly invasive lung transplantation, and palliative care. The two approved therapies are the antifibrotic agents pirfenidone and nintedanib. The efficacy of these two therapies is very similar, with several studies showing no significant difference between either drug when comparing the decline of pulmonary function and 2-year mortality rates [20,21,22]. Clinical trials have shown these two drugs can decrease the rate of decline of forced vital capacity by 50% over a year of treatment, and 2-year mortality rates after treatment initiation were roughly 36% for pirfenidone-treated patients and 39% for nintedanib-treated patients [22,23]. Lung transplants are an alternative treatment choice, but patient eligibility and accessibility of transplantation are limited. IPF patients receive 33% of lung transplants in the US, amounting to only 834 transplants each year [24,25]. For this extremely limited number of eligible patients, transplants can have a significant benefit with 5-year survival rates of approximately 50%, though there is still a considerable risk of rejection and death after transplant [26]. Palliative care in IPF is centered around symptom alleviation, particularly pulmonary rehabilitation, oxygen support, and cough management [14,23]. The high mortality rates, significant morbidities, and lack of effective and durable treatment options, highlight a significant unmet medical need for patients with IPF. Recently, the MSC-based therapies were investigated as a new therapeutic method for pulmonary fibrosis [27]. In this review, we discuss recent studies on the potential use of MSCs-CM as a therapeutic tool in pulmonary fibrosis, particularly in patients with IPF, evaluating the research efforts at the preclinical and clinical level being made in this area.

## 2. Conditioned Medium in Regenerative Medicine

Conditioned medium (CM) describes spent medium from cultured cells that contain growth factors, metabolites, and extracellular matrix proteins produced into the medium by the cultured cells [28]. Metabolites comprise of essential components like nucleotides, amino acids, and glucose, while matrix proteins include proteoglycans, fibronectin, and collagen. Contrarily, the growth factors of the CM include epidermal growth factor (EGF), interleukins, and platelet-derived growth factor (PDGF) [28]. The production of these factors by the stem cells allows the components to repair damaged tissues and cells without the stem cell itself. CM, therefore, has been described as a stem cell culture medium that contains the cultured cells’ secretome [7,29].

Since the CM of stem cells contains the soluble mediators of the secretome that are involved in the repair and regeneration of tissues and cells, it should have the regenerative properties of cell therapy, acting as cell substitutes, exerting similar therapeutic efficacy in the treatment of various disorders [30]. MSCs-CM has been shown to have beneficial impacts on bone and tissue regeneration since the secretome engages in the stimulation of different cellular duties [31]. “Secretome” contains stem cell-released mediators responsible for activating and maintaining a range of biological functions and signaling, including cell growth, differentiation, apoptosis, replication, angiogenesis, and adhesion [32]. The secretome is thought to directly mediate communication between cells or induce the surrounding cells to produce bioactive factors [32]. Secretome contains diverse types of serum proteins, angiogenic factors, growth factors, extracellular matrix protein, cytokines, hormones, extracellular matrix proteases, and lipid mediators [32]. Therefore, the secretome is the main product of stem cells that activate different biological functions involved in the treatment of various diseases and confer regenerative properties on stem cells.

MSCs secretome contains regenerative and reparative properties that lead to injury amelioration, immune modulation, or fibrosis reduction. Moreover, MSCs produce soluble proteins like chemokines and cytokines that enhance the cells’ pathological responses. The proteins include immunomodulatory impact due to the indirect and direct effects on different immune cells and response to cell or tissue injury [33]. Secretome contains cytokines and growth factors like hepatocyte growth factor (HGF), transforming growth factor-beta isoform 3 (TGF-b3), IL-10, and tumor necrosis factor-alpha (TNF-a), which moderate cell signaling and fibrogenesis processes [34]. MSCs also have paracrine effects due to the extracellular vesicles secreted from the cells that comprise an incredibly heterogeneous group of vesicles. These vesicles vary in biogenesis, size, and content. The production of extracellular vesicles creates new opportunities to deliver specific content for therapeutic applications [35]. Extracellular vesicles secreted from the MSCs carry noncoding regulator RNAs that scientists use as therapeutic agents to prompt tissue or cell regeneration [32]. Overall, the secretome of MSCs causes injury improvement, immune modulation, or reduction of fibrosis.

Thus, the possibility of using MSCs-derived CM in clinical studies may have a wide range of advantages over the use of stem cell therapy [33]. Administration of CM derived from in vitro cultures of stem cells, containing their secretory products, has been shown to repair tissues, in a similar way to stem cell therapy, and their effects may be associated with the modulation of the immune responses [36,37,38]. CM can be prepared, lyophilized, packaged, preserved, and transported easily, compared to stem cells [5]. Furthermore, there are no problems in terms of allogeneic therapy and recipient-donor matching; since no cells are administered there are no expected issues of rejection [7]. Therefore, the use of MSCs-CM appears to be a promising therapeutic strategy to produce pharmaceuticals for regenerative medicine.

The development of the most suitable techniques for quality control and efficacy tests of biopharmaceuticals founded on MSCs and their produced products stays complex. The choice of the potency test is among the leading challenges that limit the development of MSCs-CM based products. MSCs-CM-based therapeutics contain a multi-component mixture that contains multiple targets and pleiotropic impacts [32]. The main obstacle limiting the production of MSCs pharmaceuticals is the elusive nature of an action of mechanisms and the challenging choice of an active component between the secreted factors from the cells. The produced biopharmaceuticals bring out many benefits of changing cell therapy products. MSCs-CM-based pharmaceuticals signify a mix of distinct bioactive issues and multiple components produced by specific cells [39]. Therefore, MSCs-CM drugs’ production should belong to a new category based on the descriptions at the juncture of two modifiable categories: biomedical drugs and gene therapy products/advanced therapy medicinal products (CGT/ATMP).

## 3. Conditioned Medium in Pulmonary Fibrosis

Although idiopathic interstitial pneumonia, in one or another form, has been described since the late 19th century and the IPF has been known since the mid-20th century, only recently have we made progress in understanding the pathogenesis of the disease, and new antifibrotic agents have been in use, which can only attenuate symptoms and slow the progression of fibrosis [12,23,40]. It is recognized that none of the existing treatments can combat the pathogenic mechanisms of the disease, preventing fibrosis and significantly prolonging survival after diagnosis. Among the new therapeutic strategies that have been proposed, stem cell therapy is considered promising and important in the research to cure pulmonary fibrosis [40,41]. Stem cells, having the plasticity to adopt characteristics and the ability to exert functional properties of any tissue, after their migration in an injured organ, have already been considered, two decades ago, as a promising and important cell-based therapy for IPF [42]. Early studies have shown that stem cells engrafts, or intravenously injected in experimental models of pulmonary fibrosis, can migrate to the injured lung tissue and participate in tissue regeneration [43,44], and encouraged further research in the stem cell-based therapy of IPF in both animal and clinical experiments [45,46,47]. The clinical use of mesenchymal stem cell therapy has shown positive and safe treatment outcomes for patients with IPF [48,49].

Data from many clinical and preclinical studies have shown that MSCs transplantation is a possible therapeutic approach in the treatment of pulmonary fibrosis. However, the methods used to date to administer stem cells have several limitations [41]. In addition, it is not clear whether stem cells exert their regenerative property by differentiating into alveolar cells and replacing damaged tissue, or by changing the microenvironment at the site of implantation by their paracrine action. It is known that the paracrine action of MSCs with the production of secretome plays an important role in their regenerative effect [50,51]. Several studies have shown that MSCs secrete a variety of anti-inflammatory, antiapoptotic and angiogenic factors, including SDF-1, MCP-3, VEGF, and HGF, stimulating regenerative mechanisms for host cell recovery in injured tissues [5,52,53]. Khan et al have shown, using in vitro studies and a bleomycin animal model, that the secretome of lung resident mesenchymal stem cells has an antifibrotic effect and may be a novel therapeutic factor for IPF therapy [54]. Administration of MSCs-CM exerts all the beneficial effects derived from the paracrine action and secretome production from stem cell transplants, without the side effects of stem cell transplants [4]. Thus, the use of CM has been proposed as a potentially effective therapeutic strategy for the treatment of pulmonary fibrosis, and MSCs-CM has been used in experimental and clinical studies [55,56,57,58,59].

Several preclinical studies (Table 1), using in vitro and experimental animal models, have shown that MSCs-CM has an antifibrotic effect that could ameliorate pulmonary fibrosis. Cargnoni et al. have shown that the administration of human MSCs-CM to bleomycin-challenged mice significantly attenuated lung fibrosis, compared to control groups, via the reduction of fibroblast proliferation, collagen deposition and alveolar obliteration, and the decrease of lung content of IL-6, TNF-α, MIP-1α, MCP-1, and TGF-β [59,60]. Equivalent results have been presented using CM from adipose-derived stem cells in a bleomycin animal model. Treatment with CM reduced the collagen deposition and the expression of markers associated with tissue remodeling and inflammation and arrested the progression of pulmonary fibrosis [61]. Felix et al., 2019, examined the role of MSCs-CM in the emerging pathophysiological insights of modifying pulmonary fibrosis. Based on their study results, MSCs-CM led to reduced oxidative stress levels, expression of endothelin and IL-17, and recovery of lung remodeling and collagen deposition [47]. Besides that, MSCs-CM also inhibited myofibroblast activation through the limited expression of TGF-β, modulated in situ imbalance between collagen I- and collagen V-mediated IL-17 immune responses, and improved the clinical parameters of respiratory and survival rates [62].

A meta-analysis of seven studies that examined in animal models the effect of MSCs-CM in pulmonary fibrosis showed that the administration of MSCs-CM significantly decreased collagen deposition in animals with pulmonary fibrosis, and reduced the score of pulmonary fibrosis. The studies were performed on rats and mice, and they use CM from marrow-derived mesenchymal stromal cells, adipose tissue-derived mesenchymal stromal cells, and amniotic-derived mesenchymal tissue cells [55]. Subgroup analysis did not reveal any differences in the positive effect of MSCs-CM, despite the animal species, the induction of pulmonary fibrosis, or the of MSCs. The latter finding is particularly important because adipose-derived stem cells (ADSCs) are much easier to obtain and administer than other stem cell sources and could be more easily used in future studies [55].

Recently, a CM that we have prepared from cell cultures of adipose-derived MSCs (AD-MSCs), was found to inhibit the proinflammatory cytokine-induced production of various inflammatory cytokines and chemokines human, freshly isolated, pulmonary subepithelial myofibroblasts (hPSMs), and in the human epithelial lung cell line A549 [63]. This study also demonstrated that it downregulates the pro-fibrotic-induced mRNA expression of collagen Type III and the migration rate of hPSMs. In addition, using an animal experimental model of IPF, we found that our CM reduced lung tissue production of pro-inflammatory cytokines, it had an anti-fibrotic effect capable of inhibiting inflammatory and fibrotic responses in the lung, ameliorating fibrosis, and it also was safe for the animals used [56]. Overall, the CM we used seems to exert immunomodulatory properties, it was safe for the animal model, and as mentioned earlier AD-MSCs exert similar antifibrotic properties with CM of different stem cell origin [55], which makes it a plausible candidate for further development towards a treatment for IPF. There is evidence that the CM of adipose-derived-MSCs interact with NK cells, monocytes, macrophages, and effector and regulatory T cells to shift their pro-inflammatory T helper type 1 (Th1) cytokine secretion profile to an anti-inflammatory T helper type 2 (Th2) cytokine profile [64]. AD-MSCs also suppress NK cell proliferation and disrupts the migration, maturation, and antigen presentation of dendritic cells that have differentiated from monocytes [65]. CM of adipose-derived-MSCs has also been shown to stimulate the growth of new blood vessels, which is a process crucial to tissue repair, through secretion of factors such as VEGF [66]. It was also noted that MSCs combined with CM tend to repair the damaged lung tissues, which inhibit the disease’s progression [67]. Several experimental studies performed with CM have confirmed that it is safe for therapeutic use [7,68,69]. In our work, we performed an acute toxicity study using a single subcutaneous administration of 1 mg/kg of CM-ADSCs in Sprague Dawley rats without any adverse effects [56]. However, chronic toxicity, carcinogenicity, genotoxicity, and animal reproduction studies with CM are necessary to be conducted.

**Table 1:** Experimental studies on the effect of Mesenchyme Stem Cell-Derived Conditioned Medium in Idiopathic Pulmonary Fibrosis.

Study	Experimental Model	Source of CM	Outcome
Felix et al., 2019 [47]	Bleomycin model, Wistar rats	MSCs and human CM-ADSCs	Recovery of collagen deposition and lung remodeling; improvement of clinical parameters and inflammation
Filidou et al., 2021 [56]	Sprague Dawley rats; Bleomycin model, C57BL/6J mice	Human CM-ADSCs	Safety; amelioration of fibrosis; reduction of inflammation
Cargnoni et al., 2012 [59]	Bleomycin model, C57BL/6J mice	Human CM-AMTC	Reduction of fibrosis progression
Cargnoni et al., 2014 [60]	Bleomycin model, C57BL/6J mice	Human CM-AMTC	Reduction of fibrosis progression and decrease of TGF-β1, IL-6, TNF-α, MIP-1α, MCP-1 lung levels.
Rathinasabapathy et al., [61]	Bleomycin model, Sprague-Dawley rats	Rat CM-ADSCs	Reduction of collagen deposition and fibrosis progression; reduction of inflammation markers
Felix et al., 2020 [62]	Bleomycin model, Wistar rats	MSCs and human CM-ADSCs	Modulation of the in-situ imbalance between collagen I- and collagen V-mediated IL-17 immune response; Recovery of pulmonary fibrosis
Filidou et al., 2022 [63]	hPSMs; Epithelial Cells	Human CM-ADSCs	Inhibition of the proinflammatory cytokine-induced chemotaxis, the migration capacity of hPSMs and the collagen Type III mRNA expression
Shi et al., 2021 [70]	P. aeruginosa-induced lung model, C57BL/6 and BALB/c male mice	haMSC-EVs	Safety; decrease of lung inflammation and histological severity

(MSCs) conditioned medium (CM); adipose-derived stem cells (ADSCs); human amniotic mesenchymal tissue cells (AMTC); interleukin-6 (IL-6); tumor necrosis factor-α (TNF-α); macrophage inflammatory protein-1α (MIP-1α); monocyte chemoattractant protein-1 (MCP-1); and transforming growth factor-β (TGF-β); human pulmonary subepithelial myofibroblasts (hPSMs); human adipose-derived stem cells extracellular vesicles (haMSC-EVs).

To our knowledge, there are no clinical studies for using CM for treating lung injury or pulmonary fibrosis. A study, using nebulized allogenic adipose mesenchymal stromal cells-derived extracellular vesicles (haMSC-EVs) in healthy volunteers, showed that all volunteers tolerated the haMSC-EVs nebulization well, and no serious adverse events were seen. In addition, haMSC-EVs nebulization improved the survival rate in a murine lung injury model by decreasing lung inflammation and histological severity [70]. Recently, pulmonary fibrosis is one of the most severe sequelae of COVID-19 and occurs in a large proportion of patients with post-COVID-19 syndrome, while its pathogenic progression is yet to be fully elucidated [71]. Exosomes or extracellular vesicles derived from MSCs were early proposed as an alternative therapy for treating COVID-19-associated pulmonary fibrosis [72]. An early study after the onset of the COVID-19 pandemic has shown that the administration of exosomes derived from bone marrow-MSCs was safe, without adverse events, and improved clinical status and oxygenation of patients [73]. In vitro and in silico studies have shown that stem cell-derived CM reduce the cytokine storm reactions, cell death, and coagulation disturbs present in patients with both chronic inflammation diseases and viral infection by SARS-CoV-2, and could modulate the outcome in severe COVID-19 [74,75]. A recent study has shown that the MSC-EVs exert anti-inflammatory and regenerative effects directly on infected respiratory epithelial cells ameliorating the outcomes in patients with COVID-19 pneumonia and ARDS [76]. These results from clinical studies in patients with COVID-19 prove that the CM administration has a safe profile and a modulatory effect on the factors and soluble mediators, which participate in the pathogenetic mechanisms of IPF, suggesting a potential therapeutic effect.

Although the use of MSCs in chronic lung diseases, including pulmonary fibrosis, is promising and several preclinical and clinical studies support their use in the treatment of IPF, there are limitations to their use that raise safety concerns [41]. Such problems that create limitations are the selection and processing of cell grafts, the use of allogeneic versus autologous MSCs, the route of administration, ethical issues, and the rare but present possibility of carcinogenesis [53,77,78,79]. Thus, CM could be used for treating pulmonary fibrosis, mainly IPF, as a cell-free therapy with the advantages mentioned earlier compared to MCSs therapies. In addition, the intravenous administration of CM therapies makes them easier to use compared to stem cell therapies. Finally, a meta-analysis of treatments of pulmonary fibrosis, using experimental animal models, has shown that even in the chronic phases of the disease, CM treatment could control the progression of fibrosis that could be proven to be very important in the treatment of patients with IPF, mainly in the late stages of the disease [55].

## 4. Conclusions

Overall, stem cell therapy is an emerging type of treatment that helps scientists and healthcare professionals treat degenerative components of incurable illnesses using advanced biotechnology on human cells that have self-healing capabilities in the body. Treating the degenerative component of an illness overcomes the limitations of the existing drug and surgical therapy, and hence explains an increased exploration of MSCs and their CM and advancements made in this field over the years. MSCs-CM holds a promising future to cater to all the limitations with live stem cell therapy. According to the data examined in this review, MSCs-CM is an upcoming treatment option due to the nature of action and its relative safety that healthcare professionals could use to treat IPF. MSCs-CM may also prove to be a safer and more effective technique to potentially treat a wide range of acute and chronic diseases. In conclusion, in the present study we examine for first time data from recent research efforts that suggest a potential role of MSCs-CM in treating pulmonary fibrosis, and indicate that scientists are a step closer to generating a safer and more effective MSCs-based medicinal product for illnesses with an unmet need, such as IPF.

## Data Availability

No new data were created or analyzed in this study. Data sharing is not applicable to this article.
